# Mean Platelet Volume can Predict Cerebrovascular Events in Patients with Sickle Cell Anemia 

**DOI:** 10.12669/pjms.311.4104

**Published:** 2015

**Authors:** Tanju Celik, Sule Unal, Ozalp Ekinci, Cahit Ozer, Gul Ilhan, Gonul Oktay, Vefik Arica

**Affiliations:** 1 Tanju Celik, Department of Pediatrics, Mustafa Kemal University, School of Medicine, Hatay, Turkey.; 2 Sule Unal, Pediatric Hematology Clinic, Antakya State Hospital, Hatay, Turkey.; 3 Ozalp Ekinci, Ozalp Ekinci Child and Adolescent Psychiatry Clinic, Hatay, Turkey.; 4 Cahit Ozer, Department of Family Medicine, Mustafa Kemal University, School of Medicine, Hatay, Turkey.; 5 Gul Ilhan, Division of Internal Medicine Hematology Oncology, Antakya State Hospital, Hatay, Turkey.; 6 Gonul Oktay, Department of Blood Disease Center, Antakya State Hospital, Hatay, Turkey.; 7 Vefik Arica, Department of Pediatrics, Mustafa Kemal University, School of Medicine, Hatay, Turkey.

**Keywords:** Platelets, Sickle cell anemia, Thrombosis

## Abstract

**Objective:**

The purpose of this study was to determine the impact of mean platelet volume (MPV) on the frequency and severity of vaso-occlusive and cerebrovascular events in patients with sickle cell anemia (SCA).

**Methods:**

The 238 cases diagnosed with SCA were evaluated retrospectively with respect to the occurrence of painful crisis for the previous year. The incidence, severity and type of the vaso-occlusive crises of the patients with SCA between March 2010 and March 2011 were recorded. The last MPV values in patients who were free of erythrocyte transfusion for the last three months and who had no current vaso-occlusive crises were evaluated. All the patients were grouped according to the frequency of the crises for the previous year preceding the data collection. Group 1: 1 to 3 crises, Group 2: 4 to 5 and Group 3: 6 or more crises annually.

**Results:**

In accordance with the results obtained during the evaluation of the cases diagnosed with sickle-cell anemia, MPV value was found to be significantly higher in patients with cerebrovascular events. Also MPV values increased with increasing incidence of the crises (r=0.297) (p=0.001).

**Conclusion:**

One of the contributing factors for this clinical heterogeneity may be related to the MPV values in patients with sickle cell anemia. The higher MPV values may be an early predictor of future cerebrovascular events in patients with sickle cell anemia and may require close follow-up and additional measures.

## INTRODUCTION

Sickle cell anemia (SCA) is characterized by chronic hemolytic anemia and intermittent vaso-occlusive events related to a point mutation the beta-globin gene of hemoglobin molecule at the sixth amino acid (Glu6Val).^1^^-^^5^ Vaso-occlusive event results from the polymerization of deoxyhemoglobin S as a consequence there is tissue ischemia leading to acute and chronic pain as well as organ damage that can affect any organ in the body, including bones, joints, brain, eyes, liver, kidneys, and lungs. All the cellular components of the blood system are activated in SCA, MPV is a marker of platelet function, large platelets being potentially more reactive.^1^^,^^3^^,^^4^

MPV is elevated in patients with acute coronary syndrome (ACS). An increase in MPV due to smoking may also contribute to the acceleration of atherosclerosis.^6^^-^^9^ Young children more often complain of pain in their extremities, whereas older individuals more commonly experience pain in the head, chest, abdomen, and back. The major breakthrough in the field of sickle-related brain injury has been the unprecedented success of transcranial Doppler ultrasonography (TCD) to identify asymptomatic patients at high risk of stroke, coupled with chronic transfusion therapy to prevent it.^10^^,^^11^

Platelets play a crucial role in the pathogenesis of atherosclerotic complications, contributing to thrombus formation or apposition after plaque rupture.^2^^,^^7^ MPV in complete blood count analyses, indicating the size of the platelets, is higher in disorders with increased production and/or destruction of platelets including idiopatic thrombocytopenic purpura. Increased MPV has been associated with greater in vitro aggregation in response to ADP and collagen.^3^^,^^5^^,^^12^ Higher MPV levels have been identified as an independent risk factor for myocardial infarction in patients with coronary heart disease and for death or recurrent vascular events after myocardial infarction.^9^^,^^13^ Higher MPV levels have been reported in patients with vascular risk factors such as diabetes, hypercholesterolemia, and smoking and in patients with renal artery stenosis.^7^ In contrast, data regarding the association between MPV and stroke severity or stroke outcome have been controversial; on the other hand MPV has been reported as an independent predictor of the risk of stroke among individuals with a history of stroke or transient ischemic attack.^14^ The relationship between MPV and myocardial infarction or stroke indicates the platelet size as a contributory factor for clot formation through interactions of platelet function.

The purpose of this study was to determine the impact of MPV on the incidence and severity of vaso-occlusive events and cerebrovascular events in patients with SCA.

## METHODS

A descriptive, cross-sectional study was performed including 238 patients with SCA. The patient files were evaluated retrospectively. The frequency, severity and type of the vaso-occlusive crises, including acute chest syndrome, stroke or vaso-occlusive crises in the limbs between March 2010 and March 2011 were recorded. The last MPV values in patients who were free of erythrocyte transfusion for the last three months and who had no current vaso-occlusive crises were evaluated. The hematological profile of the study subjects was obtained from an automated Coulter counter (Symex XT-2000-i automated hematology analyzer, GMI). All blood samples were collected by venupencture in a potassium ethylene diamine tetra acetate (EDTA) bulb as an anticoagulant and analyzed within 15 minutes of collection.

Patients who were on anti-coagulant or anti-aggregant therapy were excluded. The severity of vaso-occlusive crisis was determined by the need of hospitalization for vaso-occlusive crises and site of vaso-occlusive crisis, so that patients who required hospitalization for crises and those who had acute chest syndrome or stroke were defined as severe. All patients were grouped according to the frequency of the crises for the previous year preceding the data collection. Group 1: 1 to 3 crises, Group 2: 4 to 5 and Group 3: 6 or more crises annually. MPV values were comparatively evaluated with the increasing number of the crises. All patients were evaluated with cranial MRI for the diagnosis of silent cerebral infarcts. The development of overt cerebrovascular event in a symptomatic patient, verified by cranial MRI, was also recorded.

***Statistical Analysis: ***Data were recorded and analyzed using SPSS 15.0. Values were shown as means ± standard deviation. ANOVA, Chi-square and Pearson’s correlation tests were used for statistical analyses.

## RESULTS

The study consisted of 238 cases diagnosed with SCA. The mean ages of these patients are 11.18±4.35 years (2 to 18 years). Of these patients, 33 (13.8%) were between 2-5 years of age, 59 (24.7%) were between 6-10 years of age and 146 (61.5%) were above 10 years of age upto 18 years-old. 118 (49.6%) of our cases were female.The gender of the patients was found to have no effect on MPV, cerebrovascular event incidence, frequency of crises and hospital admissions (p>0.05).

Splenectomy was performed in 30 (12.6 %) of these patients. The number of hidroxyurea users was 52 (21.8%). Seventy-one (29.8%) patients were admitted to hospital. Thirty-six patients out of 238 patients (15.1%) who were evaluated with cranial MRI, or who developed acute symptomatic stroke were defined to have stroke.The patients were admitted to different health care facilities in the same center, including in-patient, out-patient clinics or emergency department, with various vaso-occlusive crises episodes for 5.2±3.8 (1 to 16) times annually.

Of the SCA patients, 104 (43.7%) had 1-3 attacks (Group I), 34 (14.3%) had 4-5 attacks (Group II) and 100 (42%) had more than 6 attacks (Group III). Comparisons between the groups were done using the parameters including WBC, hemoglobin, platelets. The mean hemoglobin values were measured as 8.5±1.27 mg/dl in group I, 9.14 ±1.48 mg/dl in group II and 8.4±1.32 mg/dl in group III. We found a significant difference between groups in terms of hemoglobin levels (p=0.018). Groups I and II (p=0.042) and groups II and III were statistically different for hemoglobin values; (p=0.015) however groups I and III were similar for hemoglobin. Groups I and III were statistically significantly in terms of having different MPV values (p=0,001). Whereas, there were no significant differences between groups in terms of the other parameters. The groups were also similar for age (Table-I). The platelet counts of patients were not statistically different between the groups who were on hydroxyurea or splenectomized.

**Fig.1:**
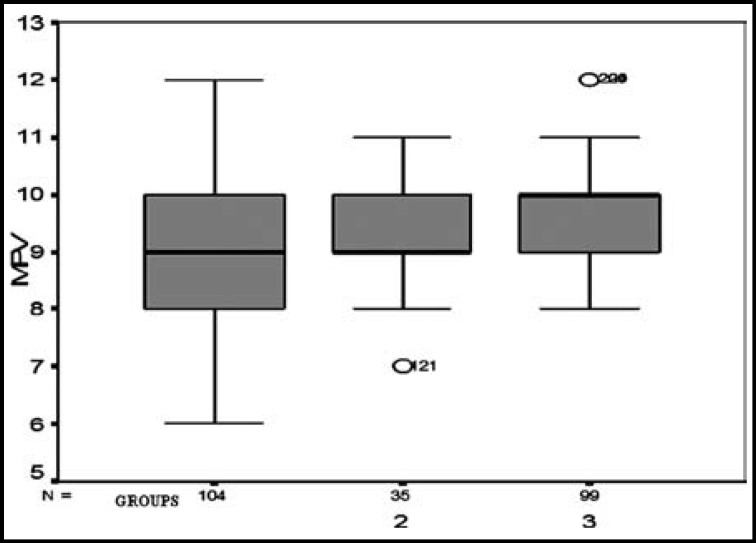
MPV values in groups 1, 2 and 3 (according to frequencies of attacks per the last year).

The MPV values of the cases were compared in terms of painful crisis. A comparison was performed according to the increased occurrence of crisis within a year. The MPV values increased with numbers of annual crises, in groups’ comparison from groups 1 to 3 (r=0.297) (p=0.001) (Table-I, Fig.1).

**Fig.2:**
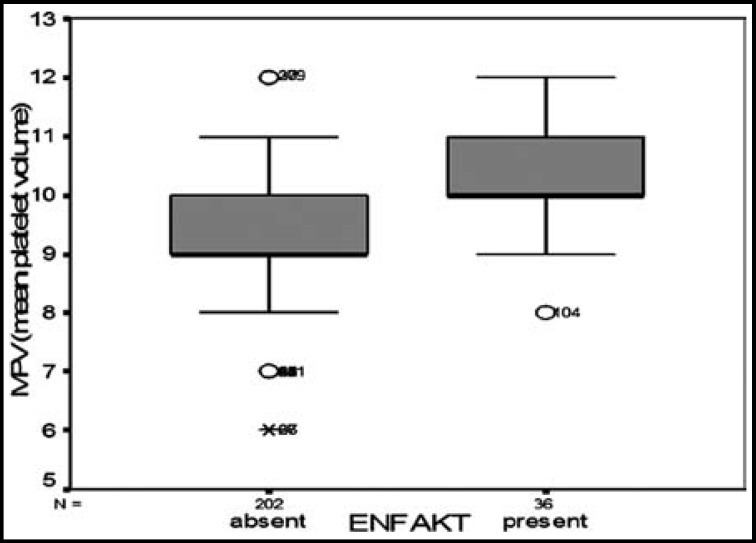
MPV values according to the development of cerebrovascular event in patients with SCA.

Out of 238 SCA patients, 36 (15.1 %) were found to have silent or symptomatic cerebral infarct by cranial MRI screening. When 36 cases with symptomatic or asymptomatic infarcts were compared with the patients without cerebrovascular events, MPV values were found as 10.22±0.95 fl vs 9.24±1.04 fl, respectively. The difference between MPV values of both groups were statistically significant (p=0.001) (Table-II, Fig.2).

As to the hospital admissions, MPV value of the patients who did not require hospitalization was 9.12±1.06 fl, whereas MPV value of the patients who were hospitalized was 10.01±0.87 fl, this difference was statistically significant (p=0.001). The MPV values of splenectomized or not-splenectomized patients and patients who were on hydroxyurea or not were statistically similar (Table-II).

## DISCUSSION

Vaso-occlusive crises and stroke are one of the major complications of SCD. Vaso-occlusion with ischemia–reperfusion injury is the central event, but the underlying pathophysiology is complex, involving a number of factors including hemolytic-associated reduction in nitric oxide bioavailability, chronic inflammation, oxidative stress, altered red cell adhesive properties, and activated white blood cells and platelets and increased viscosity.^8^ Additionally, activated platelets may also interact with abnormal red cells causing aggregation and vaso-occlusion.^15^ One of the easy methods of measuring platelet activation is MPV and this study indicates that MPV is one of the statistically significant predictors of higher vaso-oclusive crises frequency, in addition to cerebrovascular events in patients with SCA. MPV may be an easily available predictor for the prognosis in patients with acute ischemic stroke.^16^

Another study showed that there is a significant negative correlation between bleeding time and MPV in 26 patients diagnosed with ischemic heart disease. This result suggests that the higher the MPV value, the shorter the bleeding time is, i.e. the more tendencies towards thrombosis.^13^ Although, we did not measure bleeding time in our patients, MPV may have contributed to the prothrombotic state in our patients with severe sickle cell anemia, through a possible shortening of bleeding times related to the higher MPV values.

In a study comparing a group of patients with diabetes mellitus who had acute myocardial infarction with a group of patients with diabetes who did not have infarction, MPV and platelet distribution width were found as significantly higher.^17^ When the cases of severe cardiac failure were compared with the cases with moderate failure and with the ones who had never had cardiac failure, MPV was detected to be increased. The results are suggestive that the increased MPV may be due to the elevation in the platelet activity and the great platelets with increased ability of aggregation.^5^^,^^17^ We consider that the results obtained is supportive of the fact that the higher MPV value of the cases with infarction increases the probability of infarction due to the elevation of aggregation and activity of the platelets.

Most recently, a study of 89 Brazilian patients with SCD (including patients SC and Sβ-thalassemia) found an increase in the total number of platelets larger than 12 fl, especially in patients with pain crisis, but the percentage of large platelets was not increased. Their results suggest that increased interleukin serum levels probably indicate that inflammatory process is involved in the vascular-occlusive phenomenon. However, it appears that these inflammatory mediators do not have an effect on thrombopoiesis in sickle-cell-disease patients.^18^

Kenny et al measured platelet number and platelet aggregation in 16 patients with SCD on 29 occasions and were compared to both 23 healthy controls and 12 splenectomized patients who were splenectomized for any reason other than SCD. They found that platelet count and platelet aggregation were higher among both SCD patients who were autosplenectimized and patients without SCD and were splenectomized for other reasons, compared to healthy controls. They indicated that hyposplenism contributed to higher platelet activity in these patients.^19^ In this study the MPV values were similar in splenectomized or autosplenectomized patients with SCD. Some of our patients were splenectomized related to splenic sequestration crises. The rest were splenectomized related to splenomegaly induced hypersplenism. In our region there is a group of patients with SCD who have homozygous SS disease but have splenomegaly, rather than shrinkage of spleen, conflicting the commonly accepted situation. This has been reported also by some previous authors and was defined as a fibro congestive splenomegaly.^20^

Hydroxurea is a widely used agent for the prevention of vaso-occlusive crises. A previous study has reported that hydroxyurea increased hemoglobin level (8.3 vs 9.1 g/dl, *p *< 0.005), F cells (15.9% vs 37.1%) and mean corpuscular volume (82 fL vs 101 fL).^21^ In our study, hydroxyurea recievers and unreceivers were compared and, no difference was found between the groups in terms of MPV and platelet counts.

Acute symptomatic stroke is usually infarctive in children, although hemorrhagic stroke may occur, particularly in older children. Transcranial Doppler screening of patients with SCD in order to determine the risk of cerebrovascular events is highly suggested and patients with the highest time-averaged mean velocity ≥200 cm/s in the distal internal carotid artery (ICA). Its bifurcation and the middle cerebral artery were required to put on chronic transfusion to maintain the hemoglobin S level below 30% in order to prevent stroke risk.^22^^,^^23^ However, the availability of transcranial Doppler is limited in some centers and MPV may be used as a predictor of future stroke in centers where transcranial Doppler screening is unavailable. In our upcoming study, we plan to determine a cut-off value of MPV that correlates with transcranial Doppler velocity ≥200 cm/s in a larger group of patients. On the other hand, patients with higher MPV values may be suggested to be referred for transcranial Doppler evaluation as a second step from centers where transcranial Doppler is not readily available to centers where this imaging is present.

Cerebral infarction is one of the most devastating complications of SCA. In addition to the neurologic consequences, previous studies have shown that infarcts are directly related with neurocognitive impairments. Both global intellectual disabilities and specific neurocognitive impairments, including problems on attention, executive functions, memory, language and visuomotor functions, appear to be more prominent in children with cerebral infarcts. Therefore, early detection of cerebral infarcts is especially important for an improved academic achievement and related quality of life in children with SCA.^24^

**Table-I:** The relationship between hematological values and number of vaso-occlusive crises for the previous year

	**Group 1** **Mean-SD**	**Group 2**	**Group 3**	***p*** ** value**
Hemoglobin (mean ± SD)	8.5±1.27	9.1±1.48	8.4±1.32	0.018*
WBC (mean ± SD)	14.69±4.40	14.15±4.47	13.35±6.02	0.182
Platelet (mean±SD)	485±236.36	485±215.44	428±234.75	0.189
MPV (mean±SD)	9.05±1.27	9.50±1,27	9.70±1.27	0.001**
Age	11.14±4.43	10.67±4.27	11.36±4.35	0.734

* p=0,042 group 1 vs 2 and p= 0,015 group 2 vs 3

** p=0,001 group 1 vs 3.

**Table-II:** Some characteristics of patients in relation with the hematological parameters

	**WBC(x10** ^9^ **/L) (mean ± SD)**	**Hemoglobin (g/dl)** **(mean ± SD)**	**Platelet(x10** ^9^ **/L) (mean ± SD)**	**MPV(fl) (mean ± SD)**
CVE present (n=36)	13.52 ± 4.25	8.69 ± 1.54	493 ± 278.48	10.22 ± 0.95
CVE absent (n=202)	14.13 ± 5.31	8.54 ± 1.32	456 ± 224.31	9.24 ± 1.04
*p* value	0.453	0.599	0.445	0.001
Inpatient (n=71)	14.39 ± 5.19	8.53 ± 1.35	473 ± 269.94	10.01 ± 0.88
Outpatient (n=167)	14.31 ± 5.14	8.58 ± 1.36	456 ± 216.16	9.11 ± 1.05
*p* value	0.208	0.789	0.614	0.001
Hidroxyurea(+) (n=52)	13.61 ± 4.97	8.48 ± 1.43	457 ± 242.11	9.38 ± 0.88
Hidroxyurea(-) (n=186)	14.16 ± 5.22	8.59 ± 1.34	463 ± 231.10	9.38 ± 1.13
*p* value	0.491	0.588	0.872	0.987
Splenectomized (n=30)	14.10 ± 5.20	8.30 ± 1.55	442.73 ± 210.35	9.23 ± 1.00
Non-splenectomized (n=208)	14.03 ± 5.00	8.61 ± 1.32	464.55 ± 236.47	9,40±1.09
*p* value	0.947	0.243	0.604	0.383

(CVE: Cerebrovascular event; MPV: mean platelet volume)

In a different study, they studied 1,244 adult inpatients. High MPV thus appears correlated with myeloproliferative disease or thalassemia; and low MPV, with cytotoxic drugs or marrow hypoplasia.^25^

In another study, they report the results from a cross-sectional study of 216 children admitted with complaints suggestive of vaso-occlusive crisis. They observed a strong association between platelet distribution width and sickle cell disease as compared to the children who had HbAA genotype.^26^

## CONCLUSION

In the light of our findings, the measurement of MPV may be considered as an easy and effective way of screening for cerebral infarcts. Genetic, environmental, and/or technical variations may account for the reported differences in platelet size in patients with SCD. Additional studies of platelet size in humans with SCD, along with appropriate controls, are needed to clarify this issue. In conclusion, MPV is one of the factors that contribute to higher frequencies of vaso-occlusive crises and cerebrovascular events. The patients with higher MPV values need further attention for the development of cerebrovascular events.

## Authors contributions:

**Tanju Celik,** **Sule Unal,** **Ozalp Ekinci, Cahit Ozer, Gul Ilhan, Gonul Oktay and Vefik Arica** have primary responsibility for protocol development, patient screening, enrolment, data analysis and writing the manuscript.
